# Reconfigurable biconcave lens antenna based on plasma technology

**DOI:** 10.1038/s41598-023-36332-9

**Published:** 2023-06-06

**Authors:** Fatemeh Sadeghikia, Kazem Zafari, Mohammad-Reza Dorbin, Mohamed Himdi, Ali Karami Horestani

**Affiliations:** 1 Wireless Telecommunication Group, ARI, Ministry of Science, Research and Technology, Tehran, Iran; 2 Iran University of Science and Technology, Tehran, Iran; 3 Center of Excellence on Applied Electromagnetic Systems, School of ECE, College of Engineering, University of Tehran, Tehran, Iran; 4 Institute of Electronics and Telecommunications of Rennes (IETR), UMR-6164, University of Rennes 1, 35042 Rennes Cedex, France

**Keywords:** Engineering, Physics

## Abstract

This article is focused on the application of plasma technology for the development of microwave lens antennas with electronically controllable radiation gain. With this aim, the analytical background and design procedure for designing a biconcave lens based on plasma dielectric material are presented. The procedure is used to design a plasma lens antenna with a pyramidal horn feed. The effect of switching the designed lens ON and OFF on the radiation gain of the lens antenna is investigated. It is also shown that the plasma frequency of the lens can be used to dynamically adjust the radiation gain. A one-dimensional version of the proposed plasma lens operating at 10 GHz has been developed to validate the concept. Experimentally measured characteristics of a fabricated prototype of the lens antenna based on commercially available fluorescent lamps confirm the presented design procedure and numerical results. The results also show that changing the plasma frequency of the lens can be used to adjust the radiation gain of the proposed lens antenna.

## Introduction

Plasma media and its characteristics have been extensively studied throughout the recent decades for different applications, and recently have attracted increasing attention for application in communication systems^1–8^. A plasma medium with partially or fully ionized gas may act as a conductor. Plasma conductors have been widely used as the main radiators in antenna structures to achieve tunability in the operating frequency or reconfigurability in the radiation pattern or for the realization of ON/OFF switchable antennas^9–13^. Despite its advantages, the application of plasma conductors as the main radiator of an antenna results in a relatively low efficiency due to the finite conductivity of the plasma^14,15^. Alternatively, plasma structures can be used as reflectors to provide attractive functionalities such as dynamic control of radiation beamwidth^16,17^ and/or steering the direction of the beam while the antenna still has an acceptable efficiency^18–34^.

Although many studies have been conducted on the application of plasma media in their conductive mode in communication systems, there are very limited studies on the applications of plasma media in their dielectric mode , especially as a dielectric in the leaky wave antennas^35,36^ or a dielectric lens in front of a feed antenna^37–39^. However, it is well known that plasma structures are relatively good dielectrics at frequencies higher than the plasma frequency^37^. At those frequencies, the plasma refracts electromagnetic waves with a refractive index that is a function of the plasma frequency. We believe that this property can be very beneficial for the realization of reconfigurable devices for the next generations of microwave communication systems. Therefore, this study intends to demonstrate the potential of plasma media in their dielectric mode for the realization of microwave components with advanced characteristics. With this aim, the study is focused on the design of a plasma lens.

Dielectric lenses are one of the most common dielectric structures that are used to enhance the antenna gain or modify the radiation pattern, for instance by collimating an incident diverging beam to prevent it from spreading in undesired directions^40^. In almost all studies in the field of lens antennas, the constituent materials of the lens have constant electromagnetic (EM) characteristics^41–43^, and therefore, dynamic adjustment of their radiation characteristics is not possible. However, recently, it has been proved that some limited technologies, including metasurfaces^44^ and plasma dielectric materials^37–39^ have the potential to provide steerable and reconfigurable lens antennas. Utilization of the plasma for focusing purposes proves advantageous as it enables the enhancement of gain of the antenna and facilitates agile beamwidth reconfiguration without the need for physical antenna movement^37^. Although these studies prove the potential capability of the plasma materials as the lens antennas, they are still limited and more investigation is necessary for designing an efficient plasma lens antenna.

This study presents the concept and the procedure of the design of a novel lens antenna structure based on the plasma dielectric material with dynamically controllable gain. The proposed lens antenna is based on a standard pyramidal horn antenna, as a feed, and a biconcave plasma lens.

The paper is organized as follows. In “Electromagnetic waves in plasma” Section, the characteristics of plasma media and electromagnetic waves propagating in such media are briefly reviewed. In “Analytical design of a plasma lens antenna” Section, the analytical background and the design procedure of a homogeneous plasma lens are presented. The section also validates the proposed design procedure through EM simulation of a biconcave plasma lens antenna, which is designed to achieve the capability of focusing the beam in both E and H-planes. “One dimensional biconcave plasma lens antenna” Section is devoted to the validation of the concept through the design, realization, and performance measurement of a one-dimensional biconcave plasma lens antenna. The study will be concluded in “Conclusion” Section.

## Electromagnetic waves in plasma

In this section, the physical characteristics of a plasma medium and its interaction with an incident EM wave are overviewed. Plasma is a dispersive medium whose dielectric constant depends on the operating frequency. The real part of complex permittivity $\varepsilon _p$ experienced by an incident EM wave hitting a cold, homogeneous, and isotropic plasma medium is^2^ :

1 $$\begin{aligned} \Re \left( \varepsilon _p\right) =\Re \left( 1-\frac{\omega _p^2}{\omega (\omega -j\nu)}\right) =1-\frac{\omega _p^2}{\omega ^2+\nu^2} \end{aligned}$$

where $\omega$ is the angular frequency of the incident EM wave in rad/s , *v* represents the collision frequency in Hertz, which is a function of the gas pressure, and $\omega _p$ is the plasma angular frequency in rad/s, which is an intrinsic property of the plasma medium and is given by:

2 $$\begin{aligned} \omega _p=\sqrt{\frac{ne^2}{m_e \varepsilon _0}} \end{aligned}$$

In this relation, *e* and $m_e$ are respectively the electron charge and mass, *n* is the plasma density which is a function of the excitation power, and $\varepsilon _0$ is the free space permittivity.

**Figure 1:**
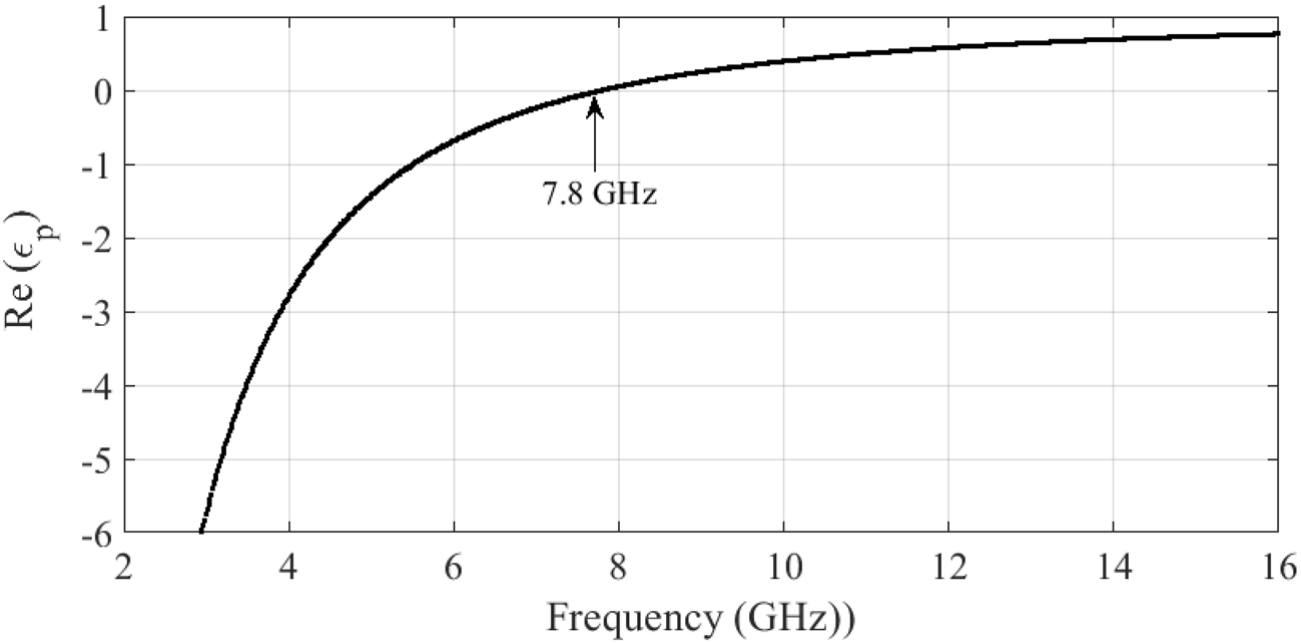
Variations of the permittivity of the plasma medium versus the frequency for the plasma frequency of 7.8 GHz and the collision frequency of $f_p/3$.

It can be seen from (1) that for an incident EM wave with a frequency greater than the plasma frequency, the plasma medium acts as a dielectric with a positive permittivity, thus permitting the propagation of the incident wave through the plasma. In contrast, at frequencies less than the plasma frequency, the plasma shows a negative permittivity, thus prohibiting the propagation of the EM wave. At frequencies much less than the plasma frequency, the plasma medium can be used as a conductor, although a not very good one. It is important to note that, since the plasma frequency $\omega _p$ can be tuned by adjusting some of the characteristics of the plasma, such as its density, the frequency band in which the plasma behaves as a dielectric or a conductor is adjustable. For instance, in Fig. 1, variations of the real part of the plasma permittivity versus the operating frequency, for a medium with the plasma frequency of 7.8 GHz and the collision frequency $\nu = f_p /3$ are shown. It is observed that at the frequencies below the plasma frequency, the plasma permittivity is negative, thus the plasma reflects the incident wave. However, at frequencies greater than 7.8 GHz, the plasma has a permittivity between 0 and 1, thus acting as a dielectric.

It is important to note that at microwave frequencies, the phase velocity $v_p$ of an EM wave propagating in a natural homogeneous unbounded dielectric medium with a relative permittivity $\varepsilon _r$ greater than one is less than the speed of light *c* in free space:

3 $$\begin{aligned} v_p=\frac{c}{\sqrt{\varepsilon _r}} \end{aligned}$$

Since the relative permittivity of a plasma medium above the plasma frequency is between zero and one, i.e., $0<\varepsilon _p< 1$, the phase velocity in such media is greater than the velocity of light in free space. This is an important feature that is taken into account in the design of plasma lenses in the next sections.

**Figure 2:**
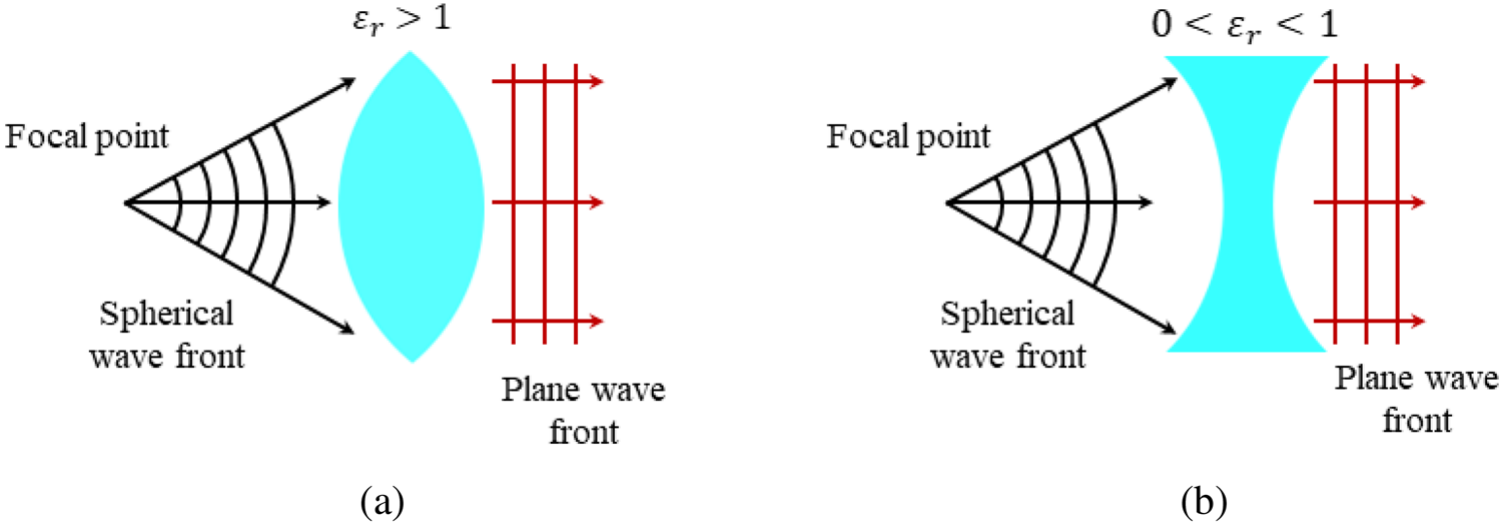
Illustrations of the geometry of different lenses for collimating a diverging beam for mediums with (**a**) $\varepsilon _r> 1$, and (**b**) $0<\varepsilon _r< 1$.

## Analytical design of a plasma lens antenna

In this section, the design procedure and the EM simulation results of a biconcave plasma lens antenna are presented. It is assumed throughout the section that the utilized plasma is an ideal homogeneous and isotropic medium.

### Design procedure of a plasma lens

**Figure 3:**
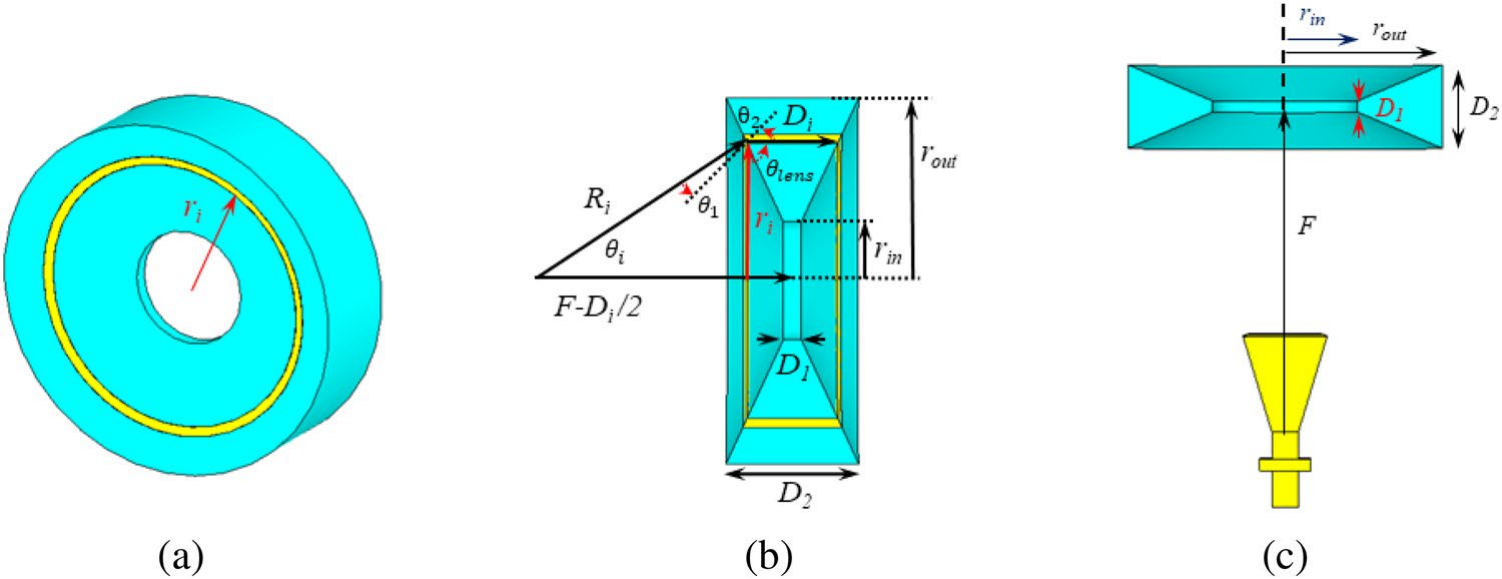
(**a**) 3-D view of a homogeneous biconcave plasma lens, (**b**) dimensions of the plasma lens, and (**c**) a homogeneous biconcave plasma lens antenna.

Generally, the shape of a dielectric lens depends on the phase velocity in the lens medium, which in turn is a function of the medium relative permittivity. It is well-known that to focus an incident beam by a lens that is formed by a conventional dielectric, the lens has to have a convex shape. It can be shown however that for a plasma medium with $0<\varepsilon _p< 1$, a concave lens is required to focus a beam, as illustrated in Fig. 2^42^. The geometry and parameters of a biconcave plasma lens are illustrated in Fig. 3a,b. In this figure, $r_{in}$ and $r_{out}$ are respectively the internal and external radii, $D_1$ and $D_2$ are respectively the minimum and maximum thicknesses, $\theta _{{{\text{lens}}}}$ is the corner angle and *F* is the focal distance of the lens. The thickness of the lens reduces from the outer diameter towards the center of the lens. As shown in the 3D view, assuming that it has a small thickness, the inner part of the lens can be removed without any adverse effect on the functionality of the lens. To collimate a spherical wavefront produced by a radiator located at the focal point *F* of the lens into an outgoing planar wavefront, each section of the wavefront passing through the lens must experience the same phase shift. Therefore, with reference to Fig. 3b, the phase shift experienced by the beam that is diverging with an angle $\theta _{i}$ must be identical to the phase shift of the beam that directly passes the center of the lens. The beam that directly passes the center of the lens travels a distance $F + \frac{D_i}{2}$ all in free space, thus experiencing a phase shift of

4 $$\begin{aligned} \phi _0=k_0\left( F+\frac{D_i}{2}\right) \end{aligned}$$

where $k_0$ is the free space wavenumber. In contrast, the diverging beam travels the path $R_i$ in free space and the path $D_i$ in the plasma medium. Therefore, when it reaches the same vertical plane as the direct beam, it has experienced a phase shift as

5 $$\begin{aligned} \phi _i=k_0(R_i+\sqrt{\varepsilon _p}D_i) \end{aligned}$$

Equating these two phase shifts gives

6 $$\begin{aligned} D_i=\frac{R_i-F}{\frac{1}{2}-\sqrt{\varepsilon _p}} \end{aligned}$$

It can be shown from the geometry that the distance $R_i$ is related to the focal distance *F* by

7 $$\begin{aligned} R_i=\sqrt{(F-\frac{D_i}{2})^2+(r_i)^2} \end{aligned}$$

Substituting $R_i$ from this equation to (6) gives the following general relation for the calculation of the thickness $D_i$ of the plasma lens at any radius $r_i$, in terms of its corresponding $r_i$, focal distance *F*, and the plasma permittivity $\varepsilon _p$ as:

8 $$\begin{aligned} D_i=\frac{\sqrt{(F-\frac{D_i}{2})^2+(r_i)^2}-F}{\frac{1}{2}-\sqrt{\varepsilon _p}} \end{aligned}$$

Substituting $\varepsilon _p$ from (1), the value of $D_i$ can be also calculated for a desired plasma frequency:

9 $$\begin{aligned} D_i=\frac{\sqrt{(F-\frac{D_i}{2})^2+(r_i)^2}-F}{\frac{1}{2}-\sqrt{1-\frac{\omega _p^2}{\omega ^2+\nu^2}}} \end{aligned}$$

As shown in the 3-D view of the biconcave lens in Fig. 3b, using Snell’s law we have:

10 $$\begin{aligned} sin(\theta _1)=\sqrt{\varepsilon _p}sin(\theta _2). \end{aligned}$$

From the figure, it is observed that

11 $$\begin{aligned} sin(\frac{\pi }{2}-\theta _i-\theta _{\text{lens}})=\sqrt{\varepsilon _p}sin(\frac{\pi }{2}-\theta _{\text{lens}}), \end{aligned}$$

in which $\theta _i$ is the angle of the incident wave from the feed point to the lens. Substituting $\theta _i$ from the geometry shown in Fig. 3b gives the following equation:

12 $$\begin{aligned} sin(\frac{\pi }{2}-tan^{-1}(\frac{r_i}{F-\frac{D_i}{2}})-\theta _{\text{lens}})=\sqrt{\varepsilon _p}sin(\frac{\pi }{2}-\theta _{\text{lens}}) \end{aligned}$$

In short, assuming a plasma frequency $f_p$, the thicknesses $D_1$ and $D_2$ can be calculated by substituting $r_i$ with the desired radius and numerically solving (9). Alternatively, fixing some of the dimensions of the lens, the required plasma frequency, and the free dimensions can be calculated to achieve a desired focal point. Substituting $D_i$ to equation (12) gives $\theta _{\text{lens}}$ for the estimation of the curvature of the lens. The curvature of the biconcave lens can be extracted by considering infinitesimal sections for the lens and calculating the length of each section. Alternatively, a lens with only one section, i.e., with a planar surface, can be designed. It can be shown that while increasing the number of sections results in a lens with curved surfaces and a slightly higher radiation gain, the design and realization of the single-section lens is much simpler, thus is used in this study. An illustration of the structure of a plasma lens antenna, involving the feeding horn antenna and the plasma lens is shown in Fig. 3c.

In summary, the presented procedure can be used to calculate the dimensions of a biconcave plasma lens based on specified plasma frequency, or the plasma permittivity. So, variations of the dielectric constant of the plasma changes the optimum dimensions of the lens.Implementation of an efficient biconcave plasma lens for different frequency bands is possible by using these equations. It is noted that the only restriction in this procedure is that the plasma frequency should be smaller than the operating frequency so that the plasma operates in its dielectric state. Moreover, to have a lens with reasonable dimensions, the permittivity of the plasma should not be too close to one. This condition is satisfied if the operating frequency is not much higher than the plasma frequency.

### Simulation of a biconcave plasma lens antenna

This section is devoted to the design and numerical simulations of a biconcave plasma lens antenna based on the procedure presented in the previous section. The numerical results are achieved using the full-wave numerical modeling software package CST Microwave Studio. The feed antenna is a standard X-band horn (LB-90-15) with 16 dBi gain and $25.8^\circ$ beamwidth in the E-plane and $25.5^\circ$ beamwidth in the H-plane. Considering the initial values $F = 300$ mm, $r_{in} =$ 60 mm, and $r_{out} =$ 120 mm, the other parameters of the plasma lens at the plasma frequency $f_p = 7.8$ GHz and the collision frequency 1.8 GHz are calculated as follows: $D_1 =$ 6.6 mm, $D_2 =$ 68.6 mm, $\theta _{\text{lens}} = 62.68^\circ$. Note that the optimum gain is achieved if the main lobe of the antenna, or in other words, the first null beamwidth (FNBW) of the feeding horn is completely covered by the lens.

**Figure 4:**
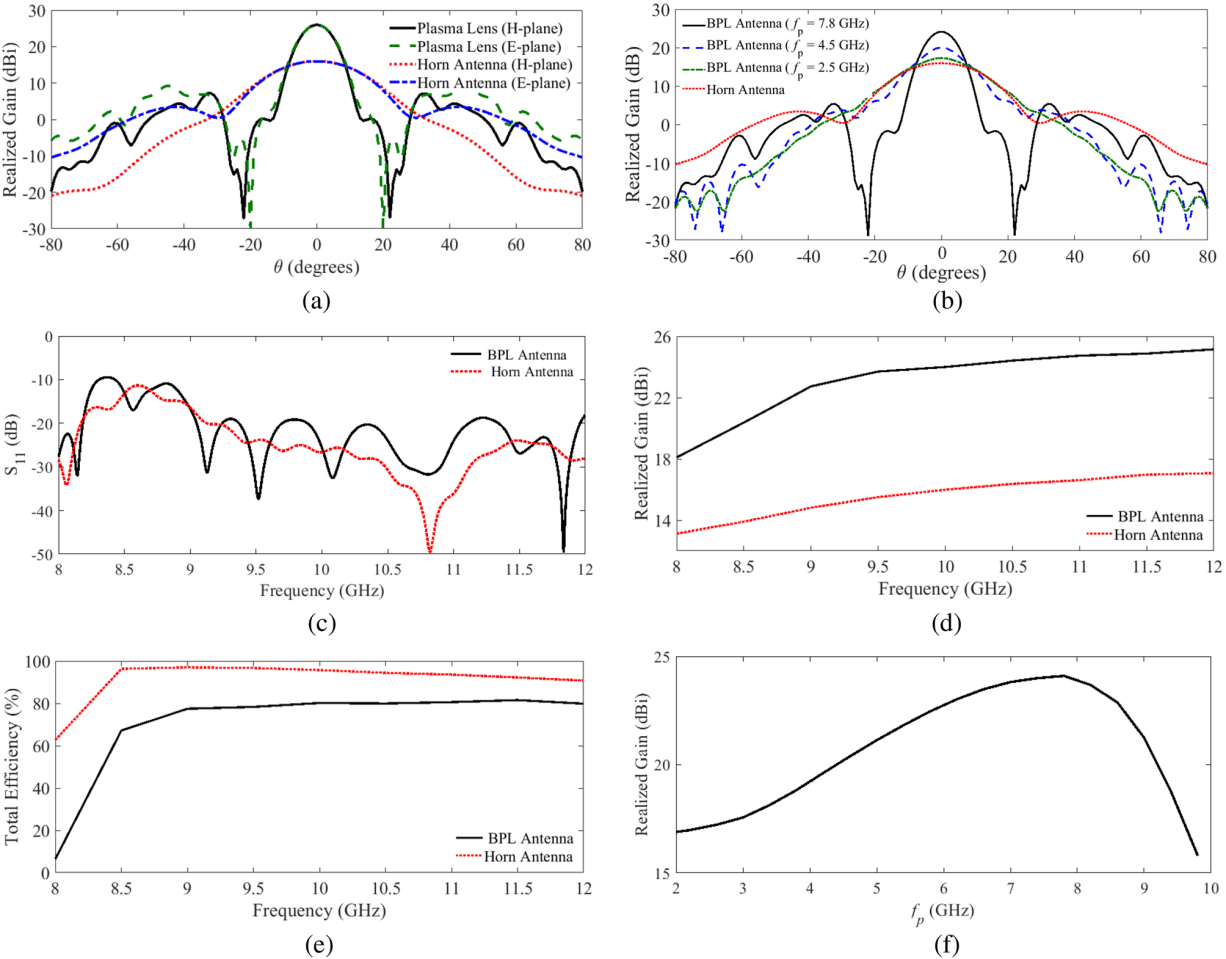
(**a**) Simulated E and H plane realized radiation gain comparison with the horn feed at 10 GHz when $f_p = 7.8$ GHz, (**b**) simulated E-plane realized radiation gain at 10 GHz for different plasma frequencies, (**c**) comparison between the simulated input reflection coefficient of the biconcave plasma lens ( BPL) antenna and that of the horn feed, (**d**) the realized gain of the BPL antenna with that of the horn feed, and (**e**) a comparison between the simulated total radiation efficiency of the BPL antenna with that of the horn feed, and (**f**) simulated radiation gain of the BPL antenna versus the plasma frequency, when the lens has been tuned to operate at $f = 10$ GHz.

In Fig. 4a, the simulated radiation gains versus $\theta$ in H- and E-planes at 10 GHz are shown. It must be noted that in these simulations the effect of the plasma container is not taken into account. The results show more than 8 dBi improvement in the radiation gain due to the plasma lens with respect to the gain of the standard feed horn. A larger increase in the radiation gain can be achieved for larger plasma lenses fed by a less directive feeding antenna located closer to the lens. Note that while the size of a plasma lens might be limited by practical issues, the design equations presented in the manuscript show no theoretical limit for the size of the lens. It is also worth noting that for the design of large-size lenses the wave amplitude also should be taken into account. The effect of the plasma frequency on the radiation gain of the biconcave plasma lens is also studied. The results of the study, which are presented in Fig. 4b, clearly show that controlling the plasma frequency from 2.5 to 7.8 GHz (while other parameters of the lens are fixed) changes the radiation gain up to 8 dBi. In short, the results show that the radiation gain of the lens antenna can be controlled by adjusting the plasma frequency. In Fig. 4c–e, the simulated return loss, the realized gain and also the total efficiency of the proposed lens antenna versus the operating frequency from $f = 8$ GHz to 12 GHz are presented and compared with those of the feeding horn. The simulated reflection coefficients of the horn antenna with and without the lens are almost identical, while it can be observed that the designed lens has improved the gain of the antenna over the whole frequency band of interest. However, as illustrated in Fig. 4f, tuning the plasma frequency in the lens controls the amount of the gain enhancement. Based on this figure, the maximum radiation gain of 24 dBi at the operating frequency of $f = 10$ GHz is achieved by tuning the plasma frequency to $f_p = 7.8$ GHz.

**Figure 5:**
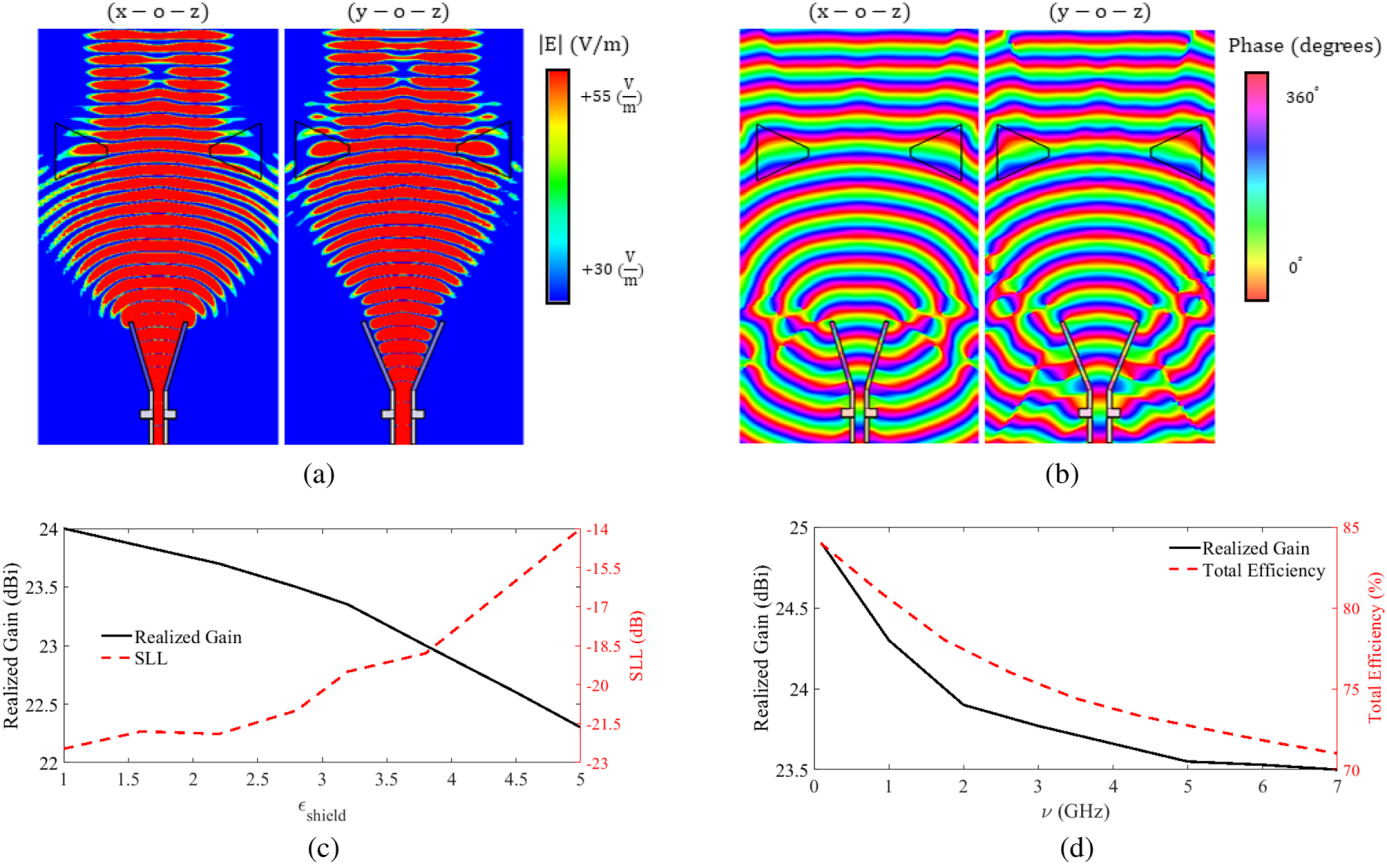
(**a**) The magnitude of the electric field at 10 GHz before and after passing the biconcave plasma lens antenna in E- and H-plans, (**b**) the phase of the electric field at 10 GHz before and after passing the biconcave plasma lens antenna in E- and H-plans, (**c**) simulated radiation gain and side-lobe levels of the biconcave plasma lens antenna versus the permittivity of the dielectric container with the thickness of $T_{shield} =1$ mm and a loss tangent of $tan(\delta ) = 0.0002$, and (**d**) simulated radiation gains and efficiency of the biconcave lens antenna versus the collision frequency of the plasma.

The magnitude and phase of the electric field before and after passing the lens, for both E- and H-plans, are illustrated in Fig. 5a,b. The figures show that the lens has collimated the beam in both planes while the phase graphs demonstrate that they are almost uniform after passing the lens. In fact, this uniformity of the phase is responsible for the increase in the directivity and gain of the structure. As mentioned earlier, the effect of the plasma container has not been taken into account in the simulation results presented so far. In practice, however, the plasma is enclosed in a dielectric container. The effect of the permittivity of a 1 mm thick container on the radiation gain and side-lobe level (SLL) of the lens antenna at $f = 10$ GHz is presented in Fig. 5c. The figure shows that the maximum radiation gain of the lens antenna corresponds to the case in which the permittivity is one, i.e., the plasma lens without container. The figure shows that increasing the permittivity decreases the gain and increases the SLL. However, it is clear from the figure that even considering a practical container, the lens improves the radiation gain of the horn. Clearly, using a low-permittivity container is preferred because it results in higher gain and lower SLL. To study the effect of the collision frequency on the performance of the lens antenna, the simulated radiation characteristics, including the radiation gain and efficiency, are presented in Fig. 5d. In General, by increasing the collision frequency, the imaginary part of the dielectric constant of the plasma increases, which in turn reduces the gain of the lens. As shown in this figure, decreasing the collision frequency from 1.8 GHz in the lens results in a higher gain and efficiency for the antenna system.

## One dimensional biconcave plasma lens antenna

The proposed concept and the design procedure of the plasma lens were verified through EM simulation of a sample plasma lens antenna in the previous section. However, the realization of such a plasma lens is not easy. One simple method to realize the designed lens is to form the desired shape by using different toroidal shape plasma structures. It can be shown that if the toroids are densely packed the EM behavior of the structure is very close to that of the original design. While this method is completely practical and cost-effective in mass production, as a low-cost proof-of-concept, a one-dimensional plasma lens using a sufficiently dense array of commercially available plasma fluorescent tubes is realized and measured in this work. In what follows, the details of the realization and experimental validation of the one-dimensional biconcave plasma lens antenna are presented.

### Design and simulation

**Figure 6:**
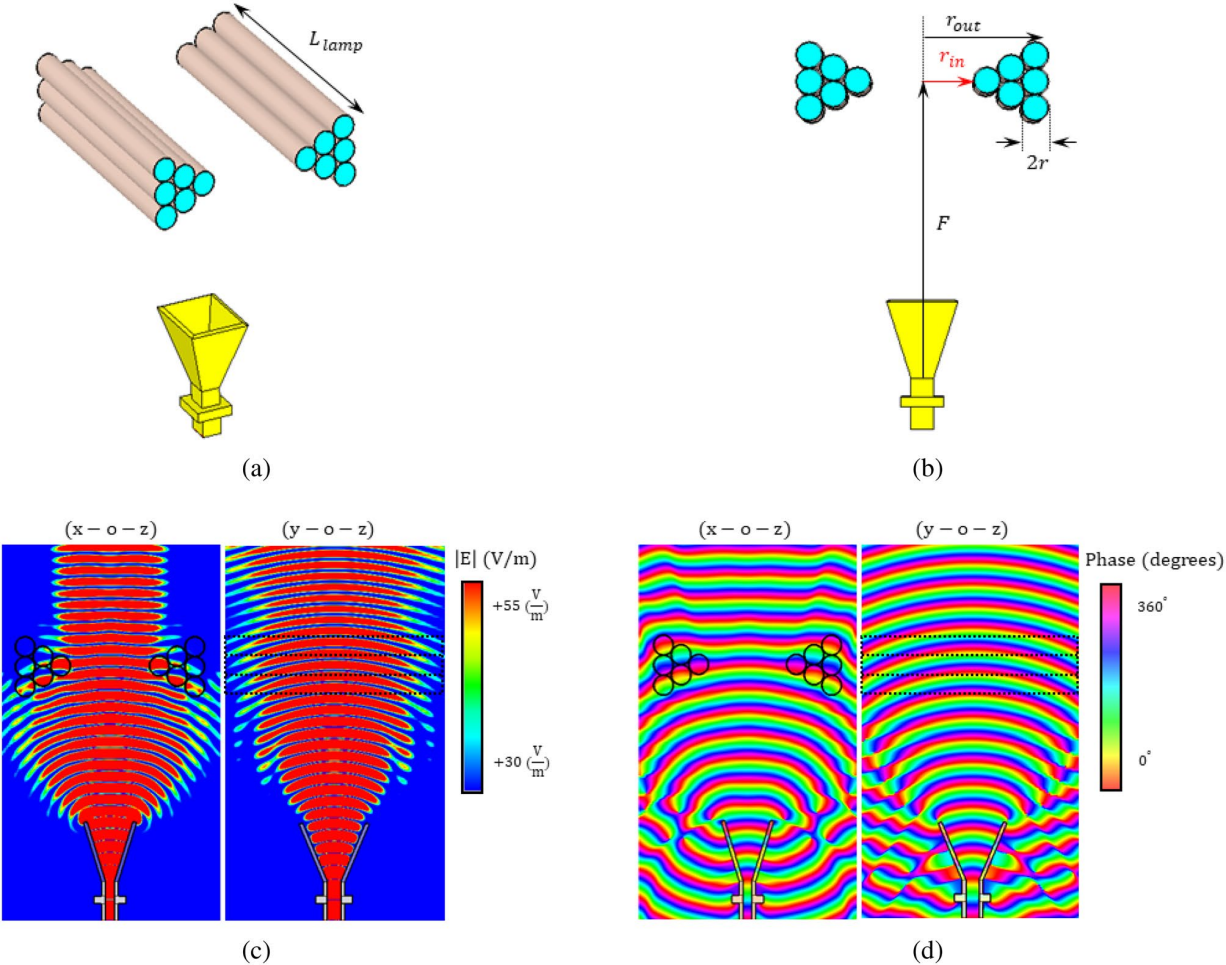
(**a**) 3-D view of the one-dimensional biconcave plasma lens antenna, (**b**) cross-section view and dimensions of the structure, (**c**) the magnitude of the electric field at 10 GHz before and after passing the one-dimensional biconcave plasma lens antenna in E- and H-plans, and (**d**) the phase of the electric field at 10 GHz before and after passing the one-dimensional biconcave plasma lens antenna in E- and H-plans.

The structure of the one-dimensional plasma lens is shown in Fig. 6a,b. As shown in the figures, the lens is formed using twelve fluorescent lamps. One can consider this structure as a cross-section of the biconcave plasma lens presented in the previous section. To facilitate the realization, the geometric scales of the lens are based on the actual dimensions of a commercially available fluorescent lamp with a length of 300 mm and a diameter of 26 mm. Dimensions and other characteristics of the lens are as follow: $f=10$ GHz, $f_{p}=7.8$ GHz, $\nu=1.8$ GHz, $F=300$ mm, $2r=26$ mm, $r_{in}=50$ mm, $r_{out}=120$ mm, $L_{lamp}=300$ mm, $T_{shield}=1$ mm, and $\varepsilon _{shield}=4.2$ . $T_{shield}$ and $\varepsilon _{shield}$ are respectively the thickness and permittivity of the dielectric container of the commercial lamps.

**Figure 7:**
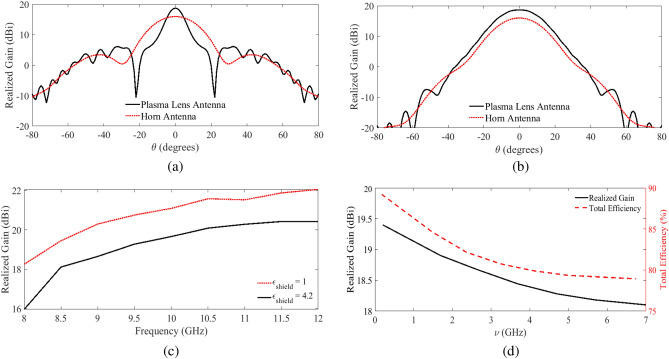
(**a**) A comparison between the radiation gain in the E-plane of the one-dimensional biconcave plasma lens antenna and the standard horn feed at 10 GHz , (**b**) a comparison between the radiation gain in the H-plane, (**c**) simulated realized gain versus the frequency for the plasma lens antenna with $\varepsilon _{shield} = 4.2$ and $\varepsilon _{shield} =1$, and (**d**) simulated realized gain and total efficiency of the lens antenna versus the collision frequency of the plasma.

Using the configuration shown in Fig. 6b , i.e., the setup in which the plasma tubes are perpendicular to the E-plane of the feeding antenna, the phase of the field is almost uniform after passing the lens, and hence the beam of the horn antenna is collimated only in the E-plane. This can be observed in Fig. 6c,d, where the snapshots of the amplitude and phase of the propagating EM wave in the E- and H-plans at $f =10$ GHz are demonstrated. The simulated radiation gain of the lens antenna in both E- and H-planes at the same frequency are compared with the horn antenna in Fig. 7a,b. The simulated gain of the lens antenna is 18.7 dBi while HPBW is around $10.2^\circ$ in the E-plane and $26.05^\circ$ in the H-plane. The results show that the proposed plasma lens focuses the beam in the E-plane which leads to a 2.7 dBi increase in the gain, while the beamwidth in the H-plane is almost untouched. Decreasing the collision frequency in the plasma lens and also a dielectric container with lower relative permittivity are two effective solutions to increase the radiated gain of the lens. For instance, in Fig. 7c, the radiation gain of the lens versus the frequency for two different cases of $\varepsilon _{shield} = 4.2$ and $\varepsilon _{shield} =1$ are compared. The results show that a decrease in the permittivity of the dielectric container to $\varepsilon _{shield} =1$, enhances the gain of the lens at least by 1.6 dBi. To study the effect of the collision frequency on the performance of the lens, the simulated radiation characteristics are presented in Fig. 7d when $\varepsilon _{shield} = 4.2$. As shown in the figure, the lower collision frequencies result in a higher gain and efficiency. As mentioned earlier, an important feature of the proposed antenna is its potential to be switched to have the original radiation pattern of the feed antenna or the modified radiation pattern with improved gain. This is a unique feature that can be achieved by switching the state of the plasma lens from OFF to ON and vice versa. This feature is important in many applications where switchable radiation gain is desired.

### Antenna assembly and measurement

**Figure 8:**
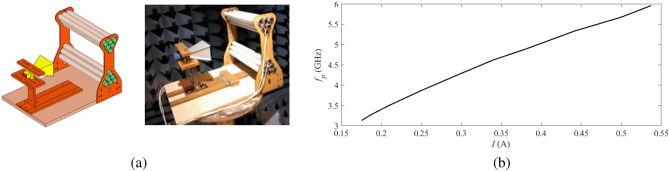
(**a**) The fabricated prototype in the anechoic chamber, and (**b**) the relation between the plasma frequency and the excitation current.

As shown in Fig. 8a, a prototype of the proposed one-dimensional plasma lens is realized by assembling the main body of the horn antenna and the lens on a structure made out of acrylic glass and wood. The structure is designed such that the position of the horn can be adjusted. This feature can help in testing lenses with different focal lengths. To energize the fluorescent lamps, a controllable DC power supply based on an electric dimmer is used in this investigation. The electric dimmer provides the capability of adjusting the plasma frequency of the lamp between 2 GHz and 7.8 GHz. The relation between the plasma frequency and the excitation current is illustrated in Fig. 8b. Measurement of the plasma characteristics of the utilized fluorescent lamp in a microwave cavity shows that for a fully ionized lamp, the plasma and collision frequencies are respectively around 7.8 GHz and 1.8 GHz.

**Figure 9:**
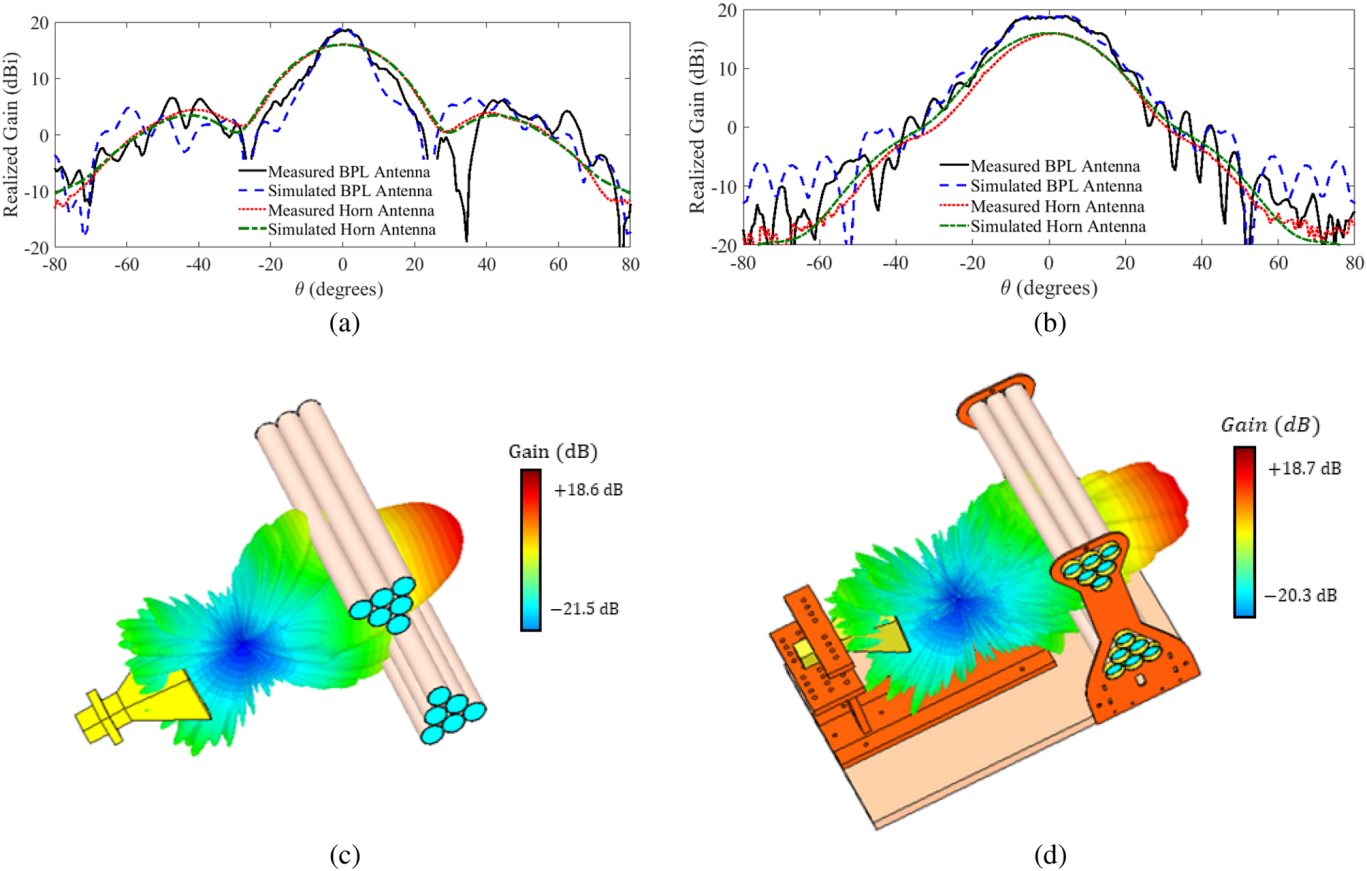
(**a**) Comparison between the measured and simulated radiation gain of the one-dimensional BPL antenna and the horn antenna at $f =10$ GHz and the plasma frequency of $f_p =7.8$ GHz in the E-plane, and (**b**) in the H-plane, (**c**) a 3-D view of the simulated radiation gain of the one-dimensional biconcave plasma lens antenna without any holding structure, and (d) with the acrylic glass and wood holder structure.

**Figure 10:**
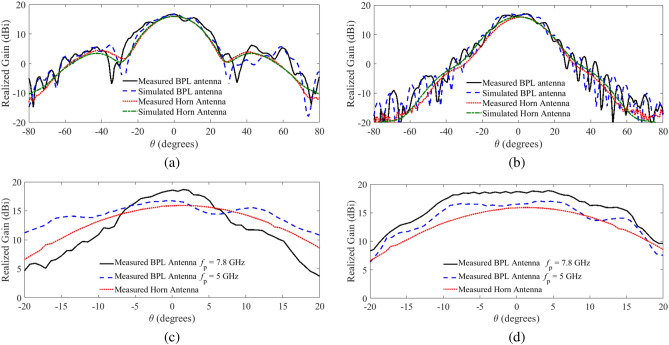
(**a**) Comparison of the radiation gain of the horn antenna to that of the one-dimensional BPL antenna at $f = 10$ GHz for the plasma frequency $f_p = 5$ GHz in the E-plane, and (**b**) in the H-plane; (**c**) Measured radiation gain of the one-dimensional BPL antenna at the frequency of 10 GHz for different plasma frequencies in the E-plane, and (**d**) in the H-plane.

The relation between the plasma frequency and the excitation current is illustrated in Fig. 8b. The measurements are performed in an anechoic chamber. The accuracy of the peak gain measurement in the chamber is better than 0.5 dBi within the frequency range of interest in this study. Figure 9a,b compare the simulated and measured radiation gains of the lens antenna in E- and H-planes. The figure also shows the simulated and measured radiation gain of the utilized horn antenna as a reference. The measured gain of the lens antenna is 18.67 dBi with a HPBW of $10.21^\circ$ in the E-plane and $25.99^\circ$ in the H-plane, which are in very good agreement with the simulation results. In short, the measured and simulated results for both planes are in good agreement, which validates the performance of the proposed plasma lens structure for improving the radiation gain. Note that, a limited increase in the SLL in E-plane is observed which is due to the holders of the lens antenna in the anechoic chamber. Also, slight changes in the H-plane pattern of the lens antenna with respect to the reference horn antenna including some ripples can be attributed to the acrylic glass holders. This is confirmed by the EM simulation of the structure with and without the holders illustrated in Fig. 9c,d. This issue can be mitigated by constructing the holder structure out of low-permittivity materials such as durable foam materials.

To experimentally validate the gain control capability of the proposed antenna, the plasma frequency is decreased from 7.8 to 5 GHz. As shown in Fig. 10a,b, the measured gain for this new plasma frequency is around 16.67 dBi which is in very good agreement with the simulated gain of 16.7 dBi for this plasma frequency. A comparison between the measured radiation gain of the one-dimensional biconcave plasma lens antenna at the frequency of 10 GHz for different plasma frequencies in the E-plane and H-plane for $f_p =7.8$ GHz, $f_p = 5$ GHz, and the horn feed is illustrated in Fig. 10c,d. The results show that the radiation gain can be controlled by adjusting the plasma frequency of the lens. In summary, the proposed plasma lens for the gain control is validated both numerically and experimentally.

## Conclusion

In this work, lens antennas based on plasma dielectric material and the associated analytical design procedure have been proposed for the first time. It has been shown that the presented method can be used for the design of biconcave lens antennas with switchable radiation gain. Using full-wave EM simulations, it was also shown that the antenna radiation gain can be controlled by controlling the plasma frequency of the lens. As a proof-of-concept, a one-dimensional prototype of the proposed lens has been designed and fabricated, and a series of measurements have been carried out for different values of the plasma frequency. The concept, design procedure, and computational results have been validated by the good agreement between the simulation and measurement results.

## Data Availability

The data produced and analyzed during the current study are available from the corresponding author on reasonable request.
