# Author Correction: Reconfigurable biconcave lens antenna based on plasma technology

**DOI:** 10.1038/s41598-023-38124-7

**Published:** 2023-07-17

**Authors:** Fatemeh Sadeghikia, Kazem Zafari, Mohammad‑Reza Dorbin, Mohamed Himdi, Ali Karami Horestani

**Affiliations:** 1 Wireless Telecommunication Group, ARI, Ministry of Science, Research and Technology, Tehran, Iran; 2 Center of Excellence on Applied Electromagnetic Systems, School of ECE, College of Engineering, University of Tehran, Tehran, Iran; 3 Institute of Electronics and Telecommunications of Rennes (IETR), UMR‑6164, University of Rennes 1, 35042 Rennes Cedex, France

Correction to: *Scientific Reports* 10.1038/s41598-023-36332-9, published online 6 June 2023

The original version of this Article contained an error in Figure 4 where the legend of the plot lines was incorrect in panel (b).

The original Figure 4 and accompanying legend appear below.

**Figure 4:**
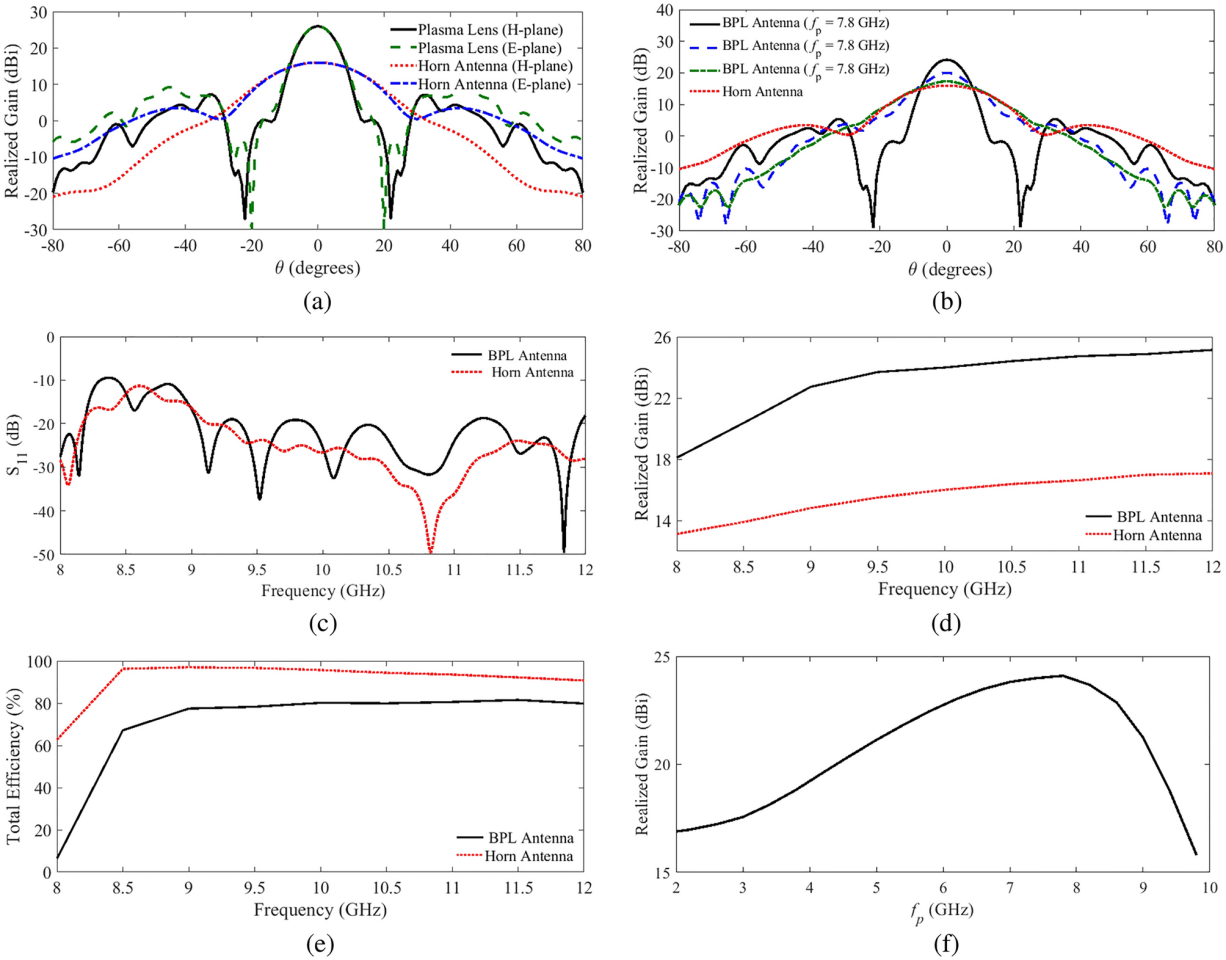
(**a**) Simulated E and H plane realized radiation gain comparison with the horn feed at 10 GHz when *f* = 7.8 GHz, (**b**) simulated E-plane realized radiation gain at 10 GHz for different plasma frequencies, (**c**) comparison between the simulated input reflection coefficient of the biconcave plasma lens (BPL) antenna and that of the horn feed, (**d**) the realized gain of the BPL antenna with that of the horn feed, and (**e**) a comparison between the simulated total radiation efficiency of the BPL antenna with that of the horn feed, and (**f**) simulated radiation gain of the BPL antenna versus the plasma frequency, when the lens has been tuned to operate at *f* = 10 GHz.

The original Article has been corrected.

