# Author Correction: Reconfigurable biconcave lens antenna based on plasma technology

**DOI:** 10.1038/s41598-025-94098-8

**Published:** 2025-03-27

**Authors:** Fatemeh Sadeghikia, Kazem Zafari, Mohammad‑Reza Dorbin, Mohamed Himdi, Ali Karami Horestani

**Affiliations:** 1 Wireless Telecommunication Group, ARI, Ministry of Science, Research and Technology, Tehran, Iran; 2 Iran University of Science and Technology, Tehran, Iran; 3 Center of Excellence On Applied Electromagnetic Systems, School of ECE, College of Engineering, University of Tehran, Tehran, Iran; 4 Institute of Electronics and Telecommunications of Rennes (IETR), UMR‑6164, University of Rennes 1, 35042 Rennes Cedex, France

Correction to: *Scientific Reports*, 10.1038/s41598-023-36332-9, published on 6 June 2023.

In the original version of this Article, Kazem Zafari was incorrectly affiliated with ‘Wireless Telecommunication Group, ARI, Ministry of Science, Research and Technology, Tehran, Iran’.

Consequently, the correct affiliation is as follows:

Iran University of Science and Technology, Tehran, Iran.

The original Article has been corrected.

